# Elucidation of FDG-PET Imaging Characteristics that Reflect the Heterogeneity within Tumors Due to Variability in Metabolic Activity: A Phantom Study

**DOI:** 10.1055/s-0044-1795105

**Published:** 2024-11-19

**Authors:** Junpei Suzuki, Keisuke Tsuda, Kazuya Koyama, Tomoya Harada, Shota Takemoto, Satoshi Kimura, Koji Murakami

**Affiliations:** 1 Department of Radiological Technology, Graduate School of Health Science, Juntendo University, Tokyo, Japan; 2 Department of Radiology, LSI Sapporo Clinic, Hokkaido, Japan; 3 Department of Radiology, Juntendo University Hospital, Tokyo, Japan

**Keywords:** silicon photomultipliers-positron emission tomography, statistical noise reduction, postprocessing filter, heterogeneity, partial volume effect

## Abstract

**Objective** Focusing on the heterogeneity within cancer lesions and revealing cancer lesions with a high signal-to-noise ratio will help improve the quality of positron emission tomography (PET) images. This study aimed to understand how glucose metabolic activity can be shown with less statistical noise using a quality assessment phantom modeled after cancer lesions with a two-layer structure and a Clear adaptive Low-noise Method (CaLM) filter.

**Materials and Methods** A National Electrical Manufacturers Association phantom with two spheres with a two-layer structure filled with 2-deoxy-2[ ^18^ F]fluoro-D-glucose (inner and outer diameters of the spheres were 13/22 and 22/37 mm, respectively; radioactivity ratio of the background [BG] to outer sphere layer was 1:4; and BG-to-inner sphere layer ratios were 1:1, 1:2, and 1:3) was evaluated. The acquisition time was set at 120 seconds, and imaging was repeated five times. The image data in photomultiplier tube (PMT)-PET were reconstructed using a time-of-flight (TOF) ordered subset expectation maximization (OSEM) algorithm (point spread function [PSF] − , iteration: 3, subset: 10) with a 4-mm Gaussian filter (GF) for normal images. Silicon photomultiplier (SiPM)-PET data were reconstructed using a TOF OSEM algorithm (PSF − , iteration: 2, subset: 12) and a 3-mm GF for normal images. The target images were reconstructed using three CaLM parameters (mild, standard, and strong). All the obtained images were investigated quantitatively, with calculation of maximum standard uptake value (SUVmax), coefficient of variation (CV), and contrast-to-noise ratio (CNR), after setting regions of interest on the lesions. Statistical analysis using the Dunnett's test compared normal images (control group) and target images (treatment group). Statistical significance was considered at *p* < 0.05.

**Results** Quantitative assessment revealed that the SUVmax of target images (standard and strong) was equivalent to that of normal images in PMT-PET, with SUVmax of 3 to 3.5 for both layers. The SUVmax in SiPM-PET was similar across all CaLM types, ranging from 3 to 4 for all spheres. The target images (standard and strong) had a significantly reduced CV and improved CNR compared with normal images.

**Conclusion** The boundary of the 22/37-mm spheres was visible with CaLM (strong) at radioactivity ratios of 1:4:1 and 1:4:2 on both scanners. For the 13/22-mm sphere boundary, visibility with CaLM (strong) was observed only with SiPM-PET, with the SUVmax equivalent to normal images. CaLM (strong) was deemed the optimal postprocessing filter for PMT-PET due to significant improvements in CV and CNR, while CaLM (standard) was suggested as the optimal filter for SiPM-PET due to excessive BG smoothing.

## Introduction

2-Deoxy-2[ ^18^ F]fluoro- d -glucose (FDG)–positron emission tomography/computed tomography (PET/CT) is extensively used to diagnose cancers, and to assess treatment effectiveness and prognosis. 1 2 Since the methods of collecting PET images and image reconstruction affect image quality, it is important to set optimal acquisition conditions and image reconstruction techniques. PET images include statistical noise, and variations in statistical noise affect clear delineations of radiotracer accumulation boundaries. 3 4 5 Conventionally, a GF is used to reduce statistical noise, although this filter also has the drawback of low contrast. 6 7 To improve this issue, in recent years, the Clear adaptive Low-noise Method (CaLM, Canon Medical Systems Corporation) filter was developed. CaLM, which is based on the nonlocal means (NLMs) method, reduces statistical noise while minimizing contrast degradation. 6 8 However, since there are no previous reports on the optimal imaging conditions for CaLM, investigation is needed to determine optimal parameter selection. The NLM filter is an edge-preserving denoising filter that takes advantage of the redundancy in images by extracting similar kernels (pixel size: 3 × 3, 5 × 5, or 7 × 7) from nonlocal regions throughout the image. It strongly smooths kernels with high similarity. 4 5 9 The filter has been proven to be efficient for noise reduction, thus increasing the signal-to-noise ratio (SNR) of the images while preserving the underlying structures. 4 5 However, Arabi and Zaidi reported that the intensity of 12-mm small spheres in the phantom was ∼60% that of 33-mm spheres in photomultiplier tube (PMT)-PET/CT. 5

In recent years, PET/CT that enhances the imaging capability for small accumulations has been developed. PET/CT using cerium-doped lutetium–yttrium orthosilicate (LYSO) scintillators and silicon photomultipliers (SiPMs) offers superior sensitivity, timing resolution, imaging capability, and statistical noise reduction compared with conventional PMT-based PET/CT scanners. 6 8 10 11 The excellent timing resolution of this SiPM-PET/CT allows for efficient acquisition of time-of-flight (TOF) information. 12 The TOF technique measures the difference in time between the detection of two annihilation photons in coincidence and allows for a more precise estimation of the annihilation location along the line of response. This leads to improved accuracy in cancer diagnosis. 13

In clinical practice, the maximum standard uptake value (SUVmax) in FDG-PET scans is frequently used as a semiquantitative indicator in the diagnosis, treatment evaluation, and prediction of prognosis of tumors. However, it does not account for the heterogeneity of glucose metabolism within the tumor lesions. 14 15 16 Tumor lesions contain multiple clones with different genomes, and the heterogeneity within the tumor lesions arises from the branching of these clones during the process of cancer evolution. 17 The concentration distribution of FDG within tumor lesions is heterogeneous due to variations in cellular proliferation, necrosis, hypoxia, and angiogenesis, making it challenging to accurately visualize local accumulation in the tumor lesions and their boundaries with surrounding normal tissues. 18 19 Moreover, the conventional spherical phantoms used in the National Electrical Manufacturers Association and the International Electrotechnical Commission (NEMA and IEC) body phantom studies do not consider the impact of heterogeneity within tumor lesions. To address this, we created a two-layer spherical phantom that simplifies mimicry of the concentration distribution within tumor lesions. Although Kazuya et al 20 reported using this two-layer spherical phantom to optimize the GF in PMT-PET, there are no prior reports on phantom experiments addressing different concentration distributions within tumor lesions using postprocessing filters or SiPM-PET, which could potentially enhance depiction capabilities.

This study aimed to elucidate the depiction characteristics of glucose metabolic activity with suppression of statistical noise, focusing on the heterogeneity concentration distributions of FDG within tumor lesions.

## Materials and Methods

### Scanner

The Celesteion PET/CT scanner (Canon Medical Systems Corporation, Otawara, Tochigi, Japan) was used for conventional PET/CT. This PET scanner is equipped with 4 × 4 mm LYSO crystals arranged in a detector ring configuration of 48 rings × 16 rows. The pixel size is 2.04 mm, with a matrix size of 208. The axial field of view (FOV) is 196 mm, allowing the acquisition of 96 PET slices per bed position. The energy window was set at 425 to 650 keV, and the coincidence time window was set at 1.6 to 3.2 nanoseconds. PET images were acquired in three-dimensional (3D) mode. For SiPM-PET/CT, the Cartesion Prime PET/CT (Canon Medical Systems Corporation) was used. This PET scanner features 4 × 4 mm LYSO crystals. The pixel size is 2.11 mm, with a matrix size of 336. The axial FOV is 271 mm, allowing the acquisition of 128 PET slices per bed position. In this study, the energy window was set at 425 to 650 keV, and the coincidence time window was set at 3.2 nanoseconds. PET images were also acquired in 3D mode. The CT device had a tube voltage of 120 kV and used automatic exposure control for the tube current, with a scan speed of 0.5 seconds. The slice thickness was 2 mm, with 0.5 mm × 16 rows for PMT-PET and 0.5 mm × 80 rows for SiPM-PET. All CT images were subjected to attenuation correction.

### Image Acquisition

**Fig. 1:**
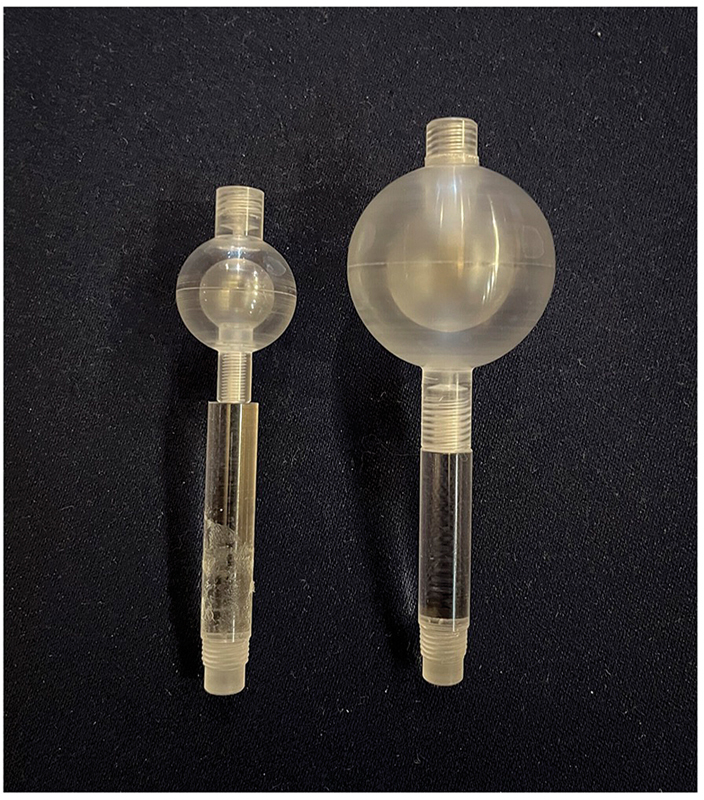
Double-sphere phantom. The inner/outer diameters of the spheres were 13/22 mm (left) and 22/37 mm (right).

**Fig. 2:**
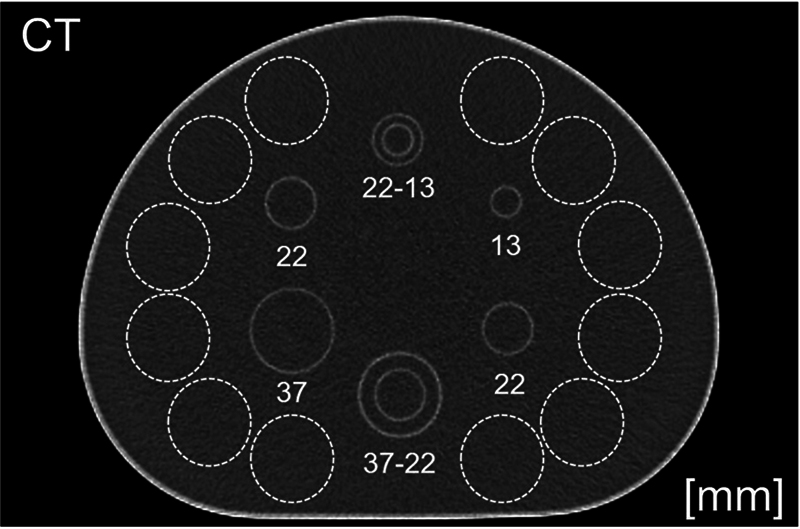
Regions of interest (ROIs) were placed on a phantom image. Twelve ROIs were placed in the background area (dotted lines) to calculate the coefficient of variation and contrast-to-noise ratio.

Images of the NEMA and IEC body phantom were acquired for evaluation. In this study, two spherical phantoms with a two-layer structure were created to simulate heterogeneous tumor uptake, as shown in Fig. 1 . Among these double spherical phantoms, the smaller phantom consisted of spheres with an outer diameter of 22 mm and inner diameter of 13 mm, while the larger phantom consisted of spheres with an outer diameter of 37 mm and inner diameter of 22 mm. The sizes and positional relationships of each sphere are illustrated in Fig. 2 . In the double-sphere phantoms, the inner layer contained a background (BG) FDG concentration equivalent to 5.3 kBq/mL, and the outer layer contained FDG at four times this concentration. Image evaluations were also performed using single-sphere phantoms as a control. In the single-sphere phantoms, the 13-mm sphere and the 22-mm sphere (which was positioned at the bottom of the body phantom) contained concentrations equivalent to the BG, while the 22-mm sphere (positioned at the top of the body phantom) and the 37-mm sphere contained FDG at four times the BG concentration, and were installed in the NEMA and IEC body phantom (BG:outer layer:inner layer = 1:4:1). The phantoms were scanned for 30 minutes in 3D-list mode, and image data were extracted at 2-minute intervals, repeated five times. The evaluations were repeated after increasing the FDG concentrations in the inner layers of the double sphere, the 13-mm single sphere, and the 22-mm single sphere positioned at the bottom to two times and three times that of the BG (BG:outer layer:inner layer = 1:4:2 and 1:4:3). For PMT-PET, the image reconstruction algorithm used was TOF 3D ordered subset expectation maximization (OSEM) (point spread function [PSF] − , iteration: 3, subset: 10). Images with a 4-mm GF (full width at half maximum [FWHM]) were used as reference images, and quantitative evaluation was performed on processed images using three CaLM filter settings (mild, standard, and strong). For SiPM-PET, the image reconstruction algorithm was TOF 3D OSEM (PSF − , iteration: 2, subset: 12), with images using a 3-mm GF (FWHM) as reference images and CaLM used for quantitative evaluation.

### Quantitative Evaluation

For quantitative evaluation, the nuclear medicine image analysis software medi + FALCON (Nihon Medi-Physics Co., Ltd., Tokyo, Japan) was used. Using this software, regions of interest (ROIs) were placed on each sphere and the BG in the image data. The SUVmax, coefficient of variation (CV), and contrast-to-noise ratio (CNR) were calculated for all images and statistically compared.

The placement of the ROIs for the BG is shown in Fig. 2 . On the central slice of the image data, eight ROIs were placed according to the size of each sphere, and SUVmax was calculated as:



where “radioactivity concentration (Bq/g)” refers to the radioactivity concentration within each sphere, “filled dose (Bq)” refers to the amount of radioactivity detected within the phantom, and “phantom weight (g)” indicates the weight of the phantom. SUVmax was defined as the maximum value of the SUV obtained using Eq. 1.

For the BG, 12 ROIs with a diameter of 37 mm were placed, and the mean and standard deviations (SDs) of the counts within the ROIs were measured for the center slice and the two slices before and after it (a total of five slices). The CV was calculated using Eq. 2, as:



where “SD” refers to the standard deviation of the pixel values within the 12 ROIs, and “average” indicates the mean of the pixel values within the 12 ROIs.

Furthermore, the maximum count of the sphere and the mean count of the central slice were measured to calculate the CNR as shown in Eq. 3, as:



where “hot sphere (maximum value)” refers to the maximum pixel value within the ROI of each sphere, “background (average value)” is the mean pixel value within the ROI placed in the BG of the central slice, and “background (SD)” indicates the standard deviation of the pixel values within the ROI placed in the BG.

Statistical analysis was performed using Dunnett's test, with the reference image as the control group and the processed images as the treatment groups. A *p* -value of less than 0.05 was considered significant.

## Results

**Fig. 3:**
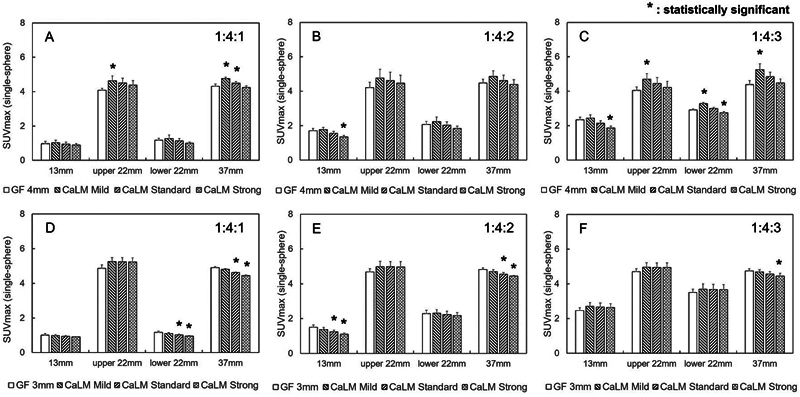
SUVmax of a single-sphere phantom. The horizontal axis represents the diameter of the spherical phantom, and the vertical axis represents the SUVmax. (A) to (C) show images acquired from PMT-PET. (D) to (F) represent images obtained from SiPM-PET. The 22-mm sphere placed at the top of the body phantom is labeled as upper 22 mm, and the 22 mm sphere placed at the bottom is labeled as lower 22 mm. PET, positron emission tomography; PMT, photomultiplier tube; SiPM, silicon photomultiplier; SUVmax, maximum standard uptake value.

Fig. 3 shows the relationship between the SUVmax of single-layer spheres and each sphere at different radioactivity concentration ratios. Except for the 13-mm sphere with a radioactivity concentration ratio of 1:4:3 in PMT-PET and the 13-mm sphere with a radioactivity concentration ratio of 1:4:2 in SiPM-PET, the SUVmax was 1 for the 13 and 22 mm spheres containing the same concentration as the BG, 2 for the double concentration, and 3 for the triple concentration. In PMT-PET, the SUVmax was 2 for the 13-mm sphere containing an FDG concentration three times that of the BG. In SiPM-PET, the SUVmax was less than 2 for the 13-mm sphere containing a concentration twice that of the BG. Additionally, all 22- and 37-mm spheres containing a concentration four times that of the BG showed an SUVmax greater than 4.

**Fig. 4:**
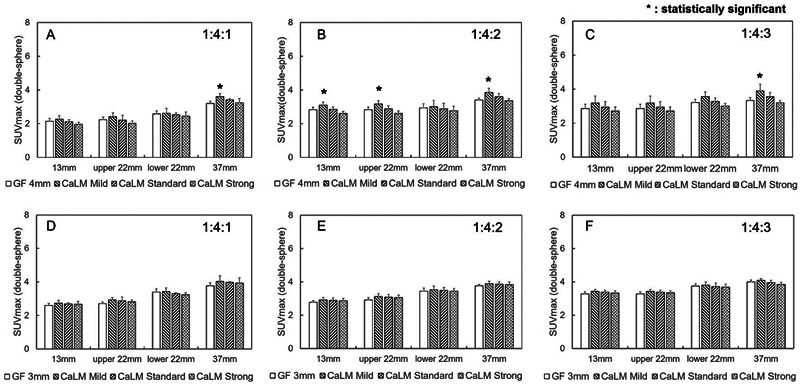
SUVmax of the double-sphere phantom. The horizontal axis represents the diameter of the spherical phantom, and the vertical axis represents the SUVmax. (A) to (C) show images acquired from PMT-PET. (D) to (F) represent images obtained from SiPM-PET. The 22-mm sphere placed at the top of the body phantom is labeled as upper 22 mm, and the 22-mm sphere placed at the bottom is labeled as lower 22 mm. PET, positron emission tomography; PMT, photomultiplier tube; SiPM, silicon photomultiplier; SUVmax, maximum standard uptake value.

Fig. 4 shows the relationship between the radioactivity concentration ratio of double-sphere phantoms, the SUVmax, and each sphere. For both scanners, when the inner layer had a concentration equivalent to BG (1:4:1) compared with the normal image, there was no significant difference in SUVmax relative to the type of filter (except for the 37-mm sphere in CaLM [mild]). When the radioactivity concentration ratio was 1:4:2, there was no significant difference between CaLM (standard and strong), but there was a significant difference for CaLM (mild) in PMT-PET. However, SUVmax for all spheres, except the 37 mm sphere in CaLM (mild), was ∼3 to 3.5. For the SiPM-PET, there was no difference in SUVmax due to the type of filter, although it showed an overall higher SUVmax compared with PMT-PET. When the radioactivity concentration ratio was 1:4:3, SUVmax for all spheres was ∼3 to 3.5.

**Fig. 5:**
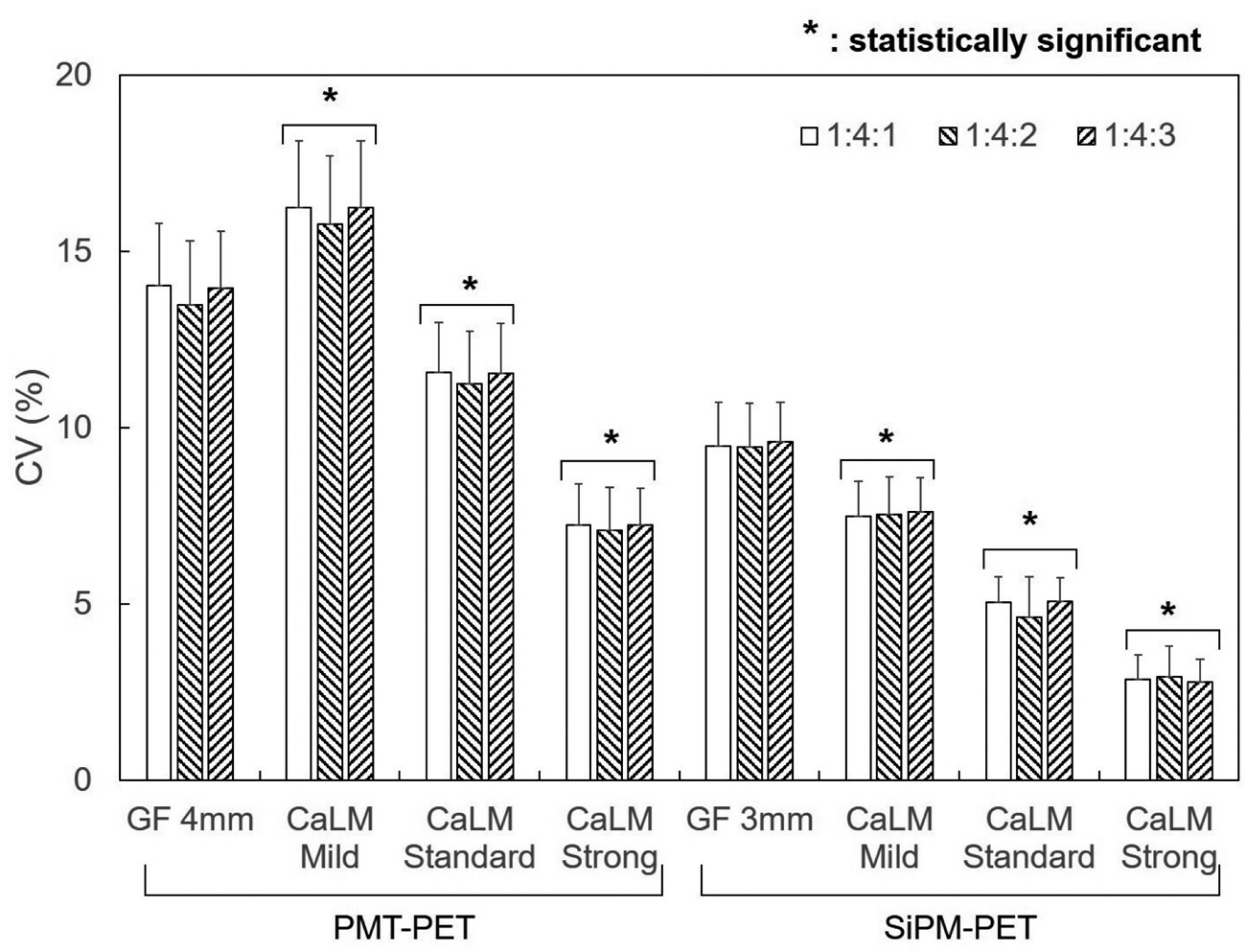
CV of BG. The horizontal axis represents the type of filter, and the vertical axis represents the CV of BG. The 22-mm sphere placed at the top of the body phantom is labeled as upper 22 mm, and the 22-mm sphere placed at the bottom is labeled as lower 22 mm. BG, background; CV, coefficient of variation; PET, positron emission tomography; PMT, photomultiplier tube; SiPM, silicon photomultiplier.

Fig. 5 shows the difference in radioactivity concentration ratios, with BG CV (%) on the vertical line and filter type on the horizontal line. In PMT-PET, CV was significantly lower in CaLM (standard and strong) compared with the normal image, with reductions of 17 and 47 to 48%, respectively ( *p* < 0.05). There were no differences due to the radioactivity concentration ratio. In contrast, for SiPM-PET, CV was significantly lower in all three CaLM types (mild, standard, and strong), showing reductions of 21, 47, and 70%, respectively ( *p* < 0.05).

**Fig. 6:**
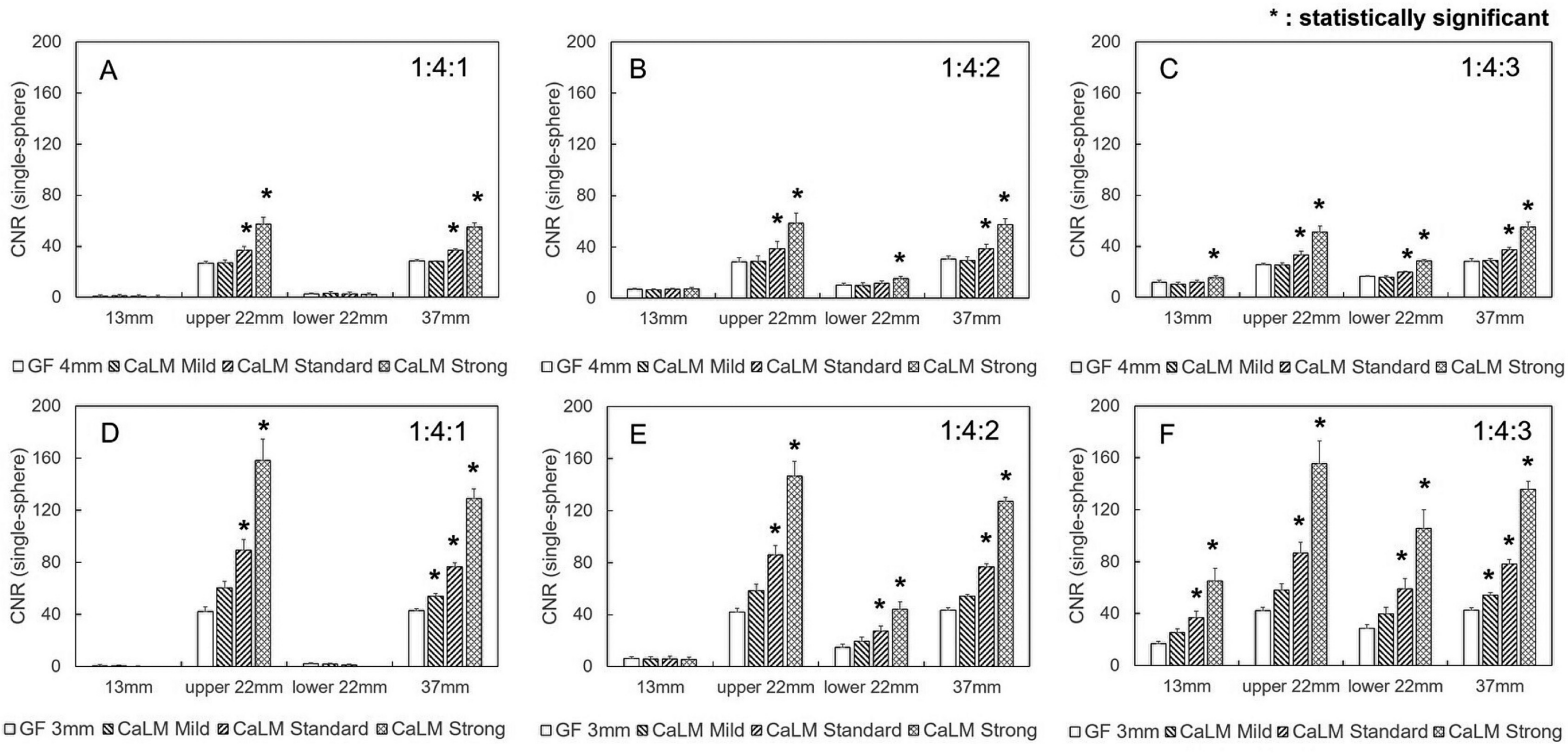
CNR of the single-sphere phantom. The horizontal axis represents the diameter of the spherical phantom, and the vertical axis represents the CNR. (A) to (C) show images acquired from PMT-PET. (D) to (F) represent images obtained from SiPM-PET. The 22-mm sphere placed at the top of the body phantom is labeled as upper 22 mm, and the 2- mm sphere placed at the bottom is labeled as lower 22 mm. CNR, contrast-to-noise ratio; PET, positron emission tomography; PMT, photomultiplier tube; SiPM, silicon photomultiplier.

Fig. 6 shows the difference in radioactivity concentration ratios, with CNR on the vertical line and sphere size on the horizontal line. In PMT-PET, CNR was significantly higher in the 13-mm sphere with CaLM (strong) at a radioactivity concentration ratio of 1:4:3. For the 22-mm sphere located below the target image, CNR was significantly higher with CaLM (strong) at a radioactivity concentration ratio of 1:4:2 and was also higher with CaLM (standard and strong) at a ratio of 1:4:3. In the 22- and 37-mm spheres with a concentration four times that of BG, CNR with CaLM (standard and strong) was improved compared with Gaussian 4 mm (FWHM). Specifically, at a radioactivity concentration ratio of 1:4:1, CNR was enhanced by 33 and 103%; at 1:4:2, by 32 and 98%; and at 1:4:3, by 31 and 98%. Additionally, in SiPM-PET, for the 22- and 37-mm spheres, CNR with CaLM (standard and strong) was improved compared with Gaussian 3 mm (FWHM). Specifically, CNR was enhanced by 95 and 238% at a radioactivity concentration ratio of 1:4:1; by 90 and 220% at 1:4:2; and by 95 and 244% at 1:4:3.

**Fig. 7:**
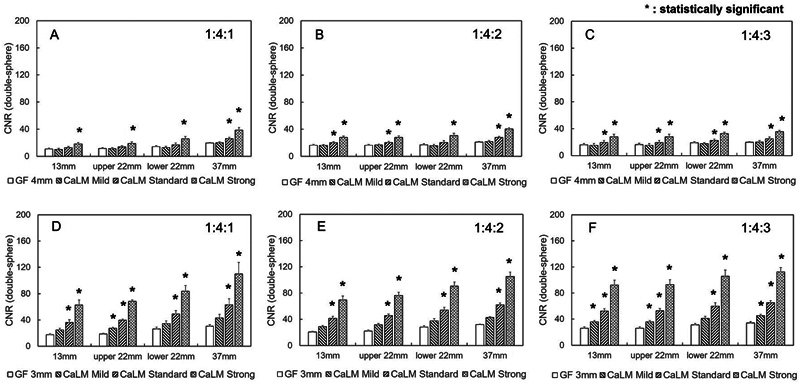
CNR of the double sphere. The horizontal axis represents the diameter of the spherical phantom, and the vertical axis represents the CNR. (A) to (C) show images acquired from PMT-PET. (D) to (F) represent images obtained from SiPM-PET. The 22-mm sphere placed at the top of the body phantom is labeled as upper 22 mm, and the 22-mm sphere placed at the bottom is labeled as lower 22 mm. CNR, contrast-to-noise ratio; PET, positron emission tomography; PMT, photomultiplier tube; SiPM, silicon photomultiplier.

Fig. 7 shows the relationship between CNR, sphere size, and filter type for the double-sphere phantom. When the radioactivity concentration ratio was 1:4:1, CNR in PMT-PET was significantly higher compared with the normal image, with increase of 24 and 78% for CaLM (standard and strong), respectively. Increases were 27 and 77%, respectively, for a ratio of 1:4:2 and 23 and 75% for a ratio of 1:4:3. The CNR in SiPM-PET also showed significantly higher values than that in PMT-PET, similar to the single-layer sphere. Specifically, CNR with CaLM (standard and strong) was significantly higher by 108 and 250%, respectively, for a radioactivity concentration ratio of 1:4:1; by 101 and 235% for a ratio of 1:4:2; and by 98 and 244% for a radioactivity concentration ratio of 1:4:3.

**Fig. 8:**
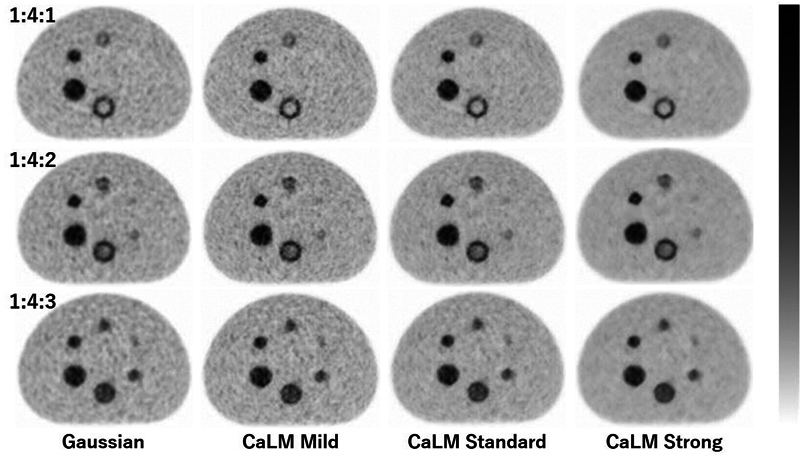
PMT-PET images. Radioactivity ratio of the BG to the outer layer to the inner layer = 1:4:1 (top row), 1:4:2 (middle row), and 1:4:3 (bottom row). From left to right, the columns represent Gaussian filter 4 mm (FWHM), CaLM (mild), CaLM (standard), and CaLM (strong). BG, background; CaLM, Clear adaptive Low-noise Method; FWHM, full width at half maximum; PET, positron emission tomography; PMT, photomultiplier tube.

Fig. 8 shows the PMT-PET images obtained 8 to 10 minutes after the start of imaging. In the target images, the boundary of the large double-sphere phantom was most clearly visible with CaLM (strong) at radioactivity concentration ratios of 1:4:1 and 1:4:2. For the boundary of the small double-sphere phantom in the target images, on the other hand, the ring shape was difficult to visualize.

**Fig. 9:**
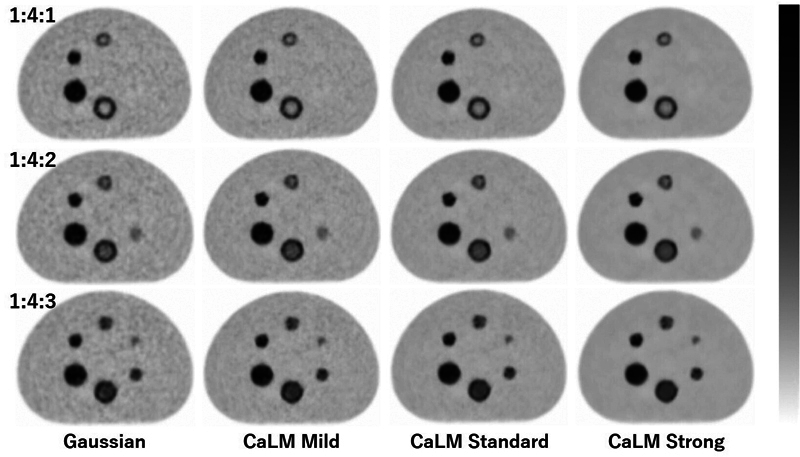
SiPM-PET images. Radioactivity ratio of the BG to the outer layer to the inner layer = 1:4:1 (top row), 1:4:2 (middle row), and 1:4:3 (bottom row). From left to right, the columns represent Gaussian filter 3 mm (FWHM), CaLM (mild), CaLM (standard), and CaLM (strong). BG, background; CaLM, Clear adaptive Low-noise Method; FWHM, full width at half maximum; PET, positron emission tomography; SiPM, silicon photomultiplier.

Fig. 9 shows the SiPM-PET images obtained 8 to 10 minutes after the start of imaging. Radioactivity concentration ratios of 1:4:1 and 1:4:2 enabled clearer visualization of the boundary of the large double-sphere phantom in the target images compared with a ratio of 1:4:3. Regarding the boundary of the small double-sphere phantom in the target images, the ring shape was not visible at a radioactivity concentration ratio of 1:4:3.

## Discussion

Tumor heterogeneity is classically associated with cellular proliferation, leading to a heterogeneous density distribution within cancer lesions. 18 19 We considered that understanding the imaging characteristics within lesions would contribute to improving cancer diagnosis in the future. In addition, the count statistics of FDG-PET images are affected by statistical fluctuations, leading to unclear shapes and boundaries of the accumulations. 3 4 5 This study aimed to elucidate the imaging characteristics of glucose metabolic activity with reduced statistical noise by focusing on the heterogeneity of FDG density distribution within cancer lesions.

Two double-sphere phantoms were created to simulate the heterogeneity within cancer lesions. Additionally, considering device dependency, evaluations were conducted using both PMT-PET and SiPM-PET. 6 8 10 11 Tsutsui et al reported that the SiPM-PET (5-mm GF) has 41% higher sensitivity and 60% shorter resolution time compared with PMT-PET (6-mm GF), with an improvement in SNR by 25% and CNR by 125%, and reduction in CV by 49%, indicating higher spatial resolution and superior statistical noise reduction. 10 Both the GF and CaLM filter are used as postprocessing filters. The conventional GF, while reducing statistical noise, has the drawback of decreasing contrast. 6 7 On the other hand, the CaLM filter can reduce statistical noise while maintaining contrast. The intensity of CaLM can be either mild, standard, or strong. 6 8 In this study, we evaluated the CaLM filter for its imaging performance in comparison to the GF.

When measuring SUV, if the size of the accumulation is less than ∼2.5 times the system spatial resolution, it is subject to partial volume effects (PVEs). 21 The system spatial resolution of PMT-PET is less than 5 mm (FWHM), 22 and the same applies to SiPM-PET. Therefore, accumulations less than 12.5 mm are likely to be underestimated. As shown in Fig. 3 , except for the 13-mm sphere, all spheres and filters in both scanners exhibited values equal to or greater than the enclosed radioactivity concentration ratio. Accumulations affected by PVE were less than 12.5 mm, and even the 13-mm sphere, which is similar in size, was considered to be influenced by PVE. As shown in Fig. 4 , SUVmax in the inner layer was equal to or greater than the enclosed radioactivity concentration ratio. In the outer layer, SUVmax was below the enclosed radioactivity concentration ratio, except for the 37-mm sphere in SiPM-PET. SUVmax exhibited the same trend across all radioactivity concentration ratios, the reason being that when calculating the counts at the boundary of the inner layer, counts from the outer layer were mixed within the same pixel (2 mm/pixel), leading to an overestimation of SUVmax in the inner layer. The ring width in the outer layer of the double-sphere phantom was 7.5 mm for the large double-sphere phantom. Therefore, compared with a standard sphere phantom (37 mm in diameter) with the same radioactivity concentration in the outer layer, SUVmax in the outer layer was considered to be underestimated due to PVE. Furthermore, in PMT-PET, the recovery coefficient (RC) for a 10-mm diameter spherical phantom is 0.49. 22 Since the ring width of the outer layer in the double-sphere phantom is less than 10 mm, the RC is likely to be further reduced. Therefore, SUVmax in the outer layer is considered to be significantly underestimated in the smaller double-sphere phantom. However, as is evident from Figs. 8 and 9 , in PMT-PET, CaLM (mild) exhibits the highest amount of statistical noise. This results in a higher presence of components considered as statistical noise in the outer layer of the double-sphere phantom. Increasing the strength of the CaLM filter reduces this statistical noise. On the other hand, in SiPM-PET, since the signal in the ring is uniform, there was no variation in SUVmax according to the strength of the CaLM filter. These phenomena occurred across all spheres. In the case of the 37-mm sphere in the outer layer of the double-sphere phantom with SiPM-PET, the decrease in SUV was mitigated, and it showed values comparable to the radioactivity concentration ratio enclosed within the sphere due to its superior sensitivity.

Fig. 5 shows that SiPM-PET had a generally lower CV compared with PMT-PET. Using PMT-PET with a radioactivity concentration ratio of 1:4:1 and applying CaLM (strong), the average BG count was 7,240 with an SD of 520. For SiPM-PET under the same conditions, the average BG count was 9,130 with an SD of 260. The CV in SiPM-PET was reduced by 31% with the GF, 53% with CaLM (mild), 56% with CaLM (standard), and 60% with CaLM (strong) compared with PMT-PET. The significant reduction in BG CV using the CaLM filter in SiPM-PET was also demonstrated in Tsutsui et al's report.

As shown in Figs. 6 and 7 , SiPM-PET improved the CNR compared with PMT-PET. In the double-sphere phantom, CaLM (standard and strong) significantly improved CNR in both scanners, regardless of the radioactivity concentration ratio. These results can be explained by the characteristics of SiPM-PET. As shown by SUVmax and CV, SiPM-PET enhances CNR due to its ability to obtain sufficient counts and reduce the BG SD. Significant differences were noted with at least CaLM (standard). In addition, the trends in CNR across the different spheres and scanners in the double-sphere phantom were consistent with those observed for SUVmax. The CNR in the inner layer was overestimated due to the mixing of outer layer counts within the same pixel. At the same time, the CNR in the outer layer was likely underestimated due to PVE and the impact of the RC.

As shown in Figs. 8 and 9 , the 13-mm sphere in the single-layer phantom was not visible even in SiPM-PET, which has superior imaging capability at a radioactivity concentration ratio of 1:4:2. There were no differences in CNR based on filter type. At a radioactivity concentration ratio of 1:4:3, accumulation was difficult to see on PMT-PET in the 13-mm sphere, and no filter-related differences in CNR were observed. However, in SiPM-PET, accumulation was clearly visible in the 13-mm sphere, and CNR improved significantly with CaLM (standard and strong). In the larger double-sphere phantom, at radioactivity concentration ratios of 1:4:1 and 1:4:2, the boundary between the inner and outer layers was most clearly visible with CaLM (strong) in PMT-PET, and the ring shape was adequately observed even with CaLM (standard) in SiPM-PET. At a radioactivity concentration ratio of 1:4:3, however, both scanners showed minimal count differences between the inner and outer layers, making it difficult to distinguish the boundary. For the smaller double-sphere phantom, PMT-PET images showed limitations in imaging capability for all radioactivity concentration ratios. In contrast, SiPM-PET was able to visualize the ring shape at ratios of 1:4:1 and 1:4:2. At a ratio of 1:4:3, even with the superior sensitivity of SiPM-PET, identifying the ring shape remained challenging.

Based on these observations, using the CaLM rather than the GF is crucial for effective, high-quality visualization of cancer lesions, with appropriate parameter selection for both scanners being essential. Considering the quantitative evaluation and PET images, CaLM (strong) is recommended for PMT-PET, whereas for SiPM-PET, although CaLM (strong) can be used, it is advisable to use CaLM (standard) in clinical settings to avoid excessive smoothing of the BG.

However, a limitation of this study is that the double-sphere phantom designed to simulate cancer lesions had a simplified structure based on existing models. In actual cancer lesions, the dose distributions are more complex. 23 Furthermore, even though the double-sphere phantom is a simplified model, it did not demonstrate the expected differences in quantitative values based on the enclosed radioactivity concentration. Additionally, when there is no significant difference in radioactivity concentrations between the inner and outer layers, the ability to visualize their boundary is reduced. In the future, increasing the radioactivity concentration ratio in the outer layer of the double-sphere phantom to create a greater difference from the inner layer is expected to mitigate the limitations in visualization capability and enable more accurate quantitative evaluation.

## Conclusion

This study attempted to simulate the distribution variability within cancer lesions and aimed to acquire PET images with reduced statistical noise while maintaining the depiction of simulated lesions. SiPM-PET was superior to PMT-PET in terms of sensitivity and statistical noise reduction.

In the target images, the boundary of the large double-sphere phantom was most clearly visible with CaLM (strong) at radioactivity concentration ratios of 1:4:1 and 1:4:2 for both scanners, with SUVmax comparable to that of normal images. For the boundary of the small double-sphere phantom in the target images, only SiPM-PET with CaLM (strong) at radioactivity concentration ratios of 1:4:1 and 1:4:2 allowed for visualization of the ring shape, with SUVmax comparable to that of the normal images. In addition, the CV and CNR of CaLM (strong) were significantly improved compared with those of normal images, suggesting that CaLM (strong) is the optimal postprocessing filter for PMT-PET. On the other hand, for SiPM-PET, considering the excessive smoothing of the BG with CaLM (strong), the results suggested that CaLM (standard) is the optimal postprocessing filter. Furthermore, since the double-sphere phantom used in this study is a simplified model, future work should involve varying the radioactivity concentration ratios in the inner and outer layers to conduct more detailed quantitative evaluations.
